# Psychological distress and e-cigarette use among young Australians: An exploratory, qualitative study

**DOI:** 10.18332/tid/189395

**Published:** 2024-06-19

**Authors:** Mary-Ellen E. Brierley, Sara Gaidoni, Michelle I. Jongenelis

**Affiliations:** 1 Melbourne Centre for Behaviour Change, Melbourne School of Psychological Sciences, The University of Melbourne, Melbourne, Australia

**Keywords:** e-cigarettes, mental health, psychological distress, young adults

## Abstract

**INTRODUCTION:**

Emerging research suggests an association between psychological distress and e-cigarette use. However, our understanding of young adults’ experiences of this relationship is limited. We explored young adults’ experiences of psychological distress and e-cigarette use.

**METHODS:**

We conducted semi-structured interviews with 18- to 24-year-old university students (n=13; 77% female; mean age=21.5 years) based in Victoria, Australia. Interviews were conducted May–June 2023. Data were subject to reflexive thematic analysis.

**RESULTS:**

Individuals reported that they or their friends had initiated e-cigarette use to manage their psychological distress (e.g. low mood, social isolation, stress). Immediate stress reduction following use and subsequent nicotine dependence appeared to maintain use.

**CONCLUSIONS:**

A reciprocal relationship likely exists between psychological distress and e-cigarette use whereby psychological distress contributes to use initiation and use maintains distress. Public campaigns and health services should provide: 1) education on adaptive coping/stress management strategies and the signs of nicotine dependence, and 2) support to manage nicotine dependence and the psychological distress that can arise from addiction.

## INTRODUCTION

Young adults (aged 18–24 years) comprise the largest and fastest-growing group of e-cigarette users in Australia^1^. In 2022–2023, approximately half reported having tried e-cigarettes, while 20% reported being current users of the devices. These figures significantly increased from those observed in 2019 (26% ever use and 5% current use)^1^.

Young adulthood is a developmental period characterized by: 1) identity exploration and formation, 2) increased independence from family, and 3) greater reliance on peers and broader society to inform one’s views and sense of self ^2^. This period also exposes individuals to multiple significant life events, such as leaving the parental home, completing high school, beginning and completing tertiary studies, undertaking employment, gaining financial independence, and developing intimate relationships^3^. These life events often cause stress and uncertainty, with mental health disorders among young adults common and increasing in prevalence^4,5^. The use of maladaptive coping strategies is higher among young adults relative to older cohorts^6^, with the use of substances such as alcohol to cope with life stressors previously identified among young adults^7,8^. It is reasonable to suggest that e-cigarettes are also being used as coping mechanisms, especially given advertising that portrays vaping as ‘calming’, ‘mood-boosting’, and ‘relaxing’^9^. Supporting this proposition, findings from previous research suggest a relationship between mental health and e-cigarette use in young adults, with longitudinal work indicating that high-severity internalizing and externalizing symptoms predict initiation of e-cigarette use^10^. Cross-sectionally, use is associated with higher levels of depression^11^ and perceived stress^12^. Finally, young adults who use e-cigarettes are more likely than those who do not report a history of anxiety, post-traumatic stress disorder, low self-esteem, and high impulsivity^13^.

Despite emerging research indicating an association between psychological distress and e-cigarette use, our understanding of young adults’ experiences of this relationship and why these factors may be related is limited. To date, no studies have used qualitative methods to understand young adults’ experiences of mental health and e-cigarette use, despite such methods being uniquely positioned to provide a nuanced and detailed understanding of this relationship. The present exploratory study thus employed qualitative methodology to explore young adults’ personal or vicarious experiences of the relationship between psychological distress and e-cigarette use. We included young adults regardless of their e-cigarette/combustible cigarette use status. This allowed us to explore the views of those with lived experience, as well as those with no lived experience, but who may be witnessing use in their everyday life and whose perceptions of the link between e-cigarette use and mental health may influence their decision to initiate or abstain from using e-cigarettes in the future.

## METHODS

This qualitative study was approved by a university human research ethics committee. All participants provided written informed consent. Individual semi-structured interviews were conducted with 13 young adults aged 18–24 years (77% female; mean age = 21.5 years) in May–June 2023. Participants were recruited via posters placed in high traffic areas at a university in Victoria, Australia. Participant characteristics are shown in Table 1. Interviews were approximately 20 minutes in duration.

**Table 1:** Participant characteristics, a qualitative interview study, Melbourne, Australia, 2023 (N=13)

*Characteristics*	*n (%)*
**Age (years),** mean (SD)	21.54 (1.67)
**Gender**	
Women	10 (77)
Men	3 (23)
**E-cigarette use**	
Current or previous use	8 (62)
Never use	5 (38)
**Cigarette use**	
Current or previous use	8 (62)
Never use	5 (38)

Prior to their interview, participants completed a short survey assessing sociodemographic characteristics (e.g. gender, age) and tobacco cigarette smoking and e-cigarette use. Responses to the questions: ‘Have you ever smoked a tobacco cigarette, even just one or two puffs?’ and ‘Have you ever used an e-cigarette, even just one or two puffs?’, were used to determine the use of cigarettes and e-cigarettes. Those who responded in the affirmative were subsequently asked: ‘How often, if at all, do you currently smoke tobacco cigarettes?’ and ‘How often, if at all, do you currently use e-cigarettes that have nicotine in them?’. Response options were daily, weekly (but not daily), monthly (but not weekly), and less often than monthly. Those who responded daily, weekly, or monthly were considered current smokers or current e-cigarette users.

Interviews were conducted by one researcher. We adopted an iterative approach to the interviews whereby the interviewer’s method remains flexible, allowing discoveries made throughout the interviewing process to be explored^14^. All interviews began with the following questions: 1) ‘What do you know about e-cigarettes and e-cigarette use (also known as vapes and vaping)?’; and 2) ‘What has been your experience with vapes and vaping?’. Given the link between e-cigarette use and mental health arose spontaneously in response to these questions in early interviews, participants were prompted to speak about their experiences with e-cigarette use and mental health in subsequent interviews where this theme was not raised spontaneously. An example question exploring this theme included: ‘Do you think that young people use vapes as a form of stress relief ?’.

### Data analysis

Interviews were audio recorded by the interviewer and subsequently transcribed verbatim by an independent and ISO-accredited transcription agency. Transcripts were imported into NVivo for coding and analysis. We took a data-driven, inductive approach to thematic analysis^15^, whereby we identified topics relating to the relationship between mental health and e-cigarette use. Consistent with this approach, one researcher undertook the iterative data analysis process, which included data familiarization, code and theme development, and refinement.

Data were initially organized into codes based on whether the content featured a mental health theme. Content specific to mental health was then analyzed, with data organized into the following identified topics: 1) mental health reasons for initiation of e-cigarette use, 2) mental health reasons for continued e-cigarette use, and 3) dependence/addiction. Given the content’s similarity, the first two codes were merged during the theme refinement process. Child codes within this merged parent code included ‘general distress’, ‘perceived stress and stress reduction’, ‘social isolation’, and ‘insecurity/self-esteem’. Child codes within the dependence/addiction parent code initially included ‘addiction’ and ‘withdrawal’. These codes were merged, given the similarity of content. MB consulted authors MJ and SG in order to review and refine the coding hierarchy, thus ensuring the identified themes accurately reflected the nature of the data.

Illustrative quotes are presented in the results section. These are followed by the participant’s demographic characteristics [age, gender (W = Woman; M = Man), and vaping and smoking status (V/S = those who currently vape/smoke or have previously vaped/smoked; NV/NS = those who have never vaped/smoked)].

## RESULTS

**Figure 1:**
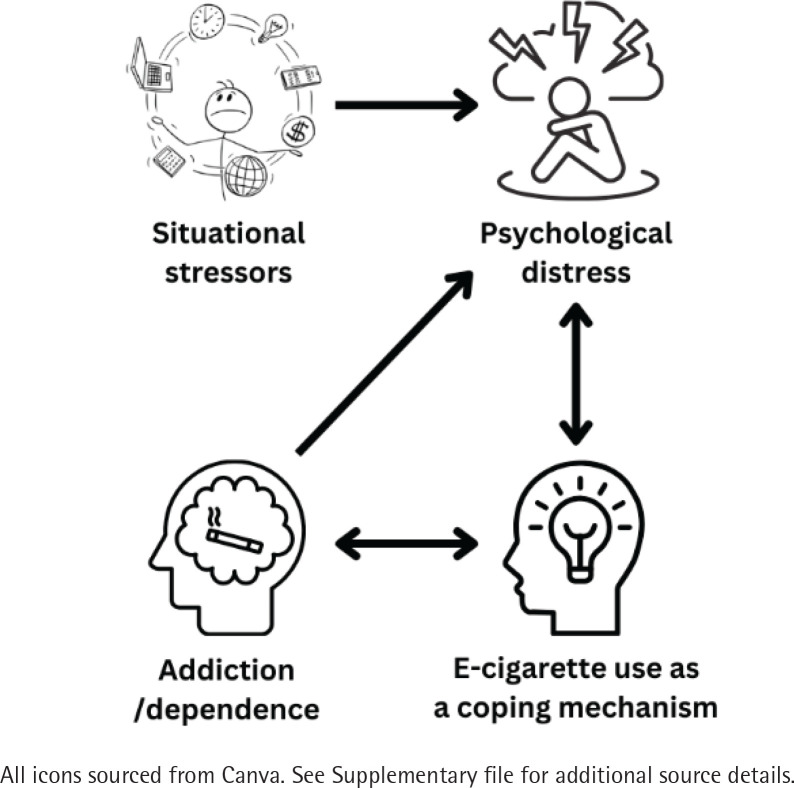
Summary of the main themes identified in the data, Melbourne, Australia, 2023 (N=13)

Seven participants spontaneously discussed their experiences of mental health and e-cigarette use, and six participants were prompted specifically questioned by the interviewer on the topic. Two key themes emerged from the data: 1) mental health-related reasons for initiation and continued use of e-cigarettes, and 2) experiences of addiction. The inter-relationships between these themes are illustrated in Figure 1.

### Mental health-related reasons for initiation and continued use of e-cigarettes

Most participants spoke of their personal experiences with or vicarious knowledge of emotional stressors preceding the initiation of e-cigarette use. Participants noted using e-cigarettes – or seeing e-cigarettes being used by friends and peers – to manage psychological distress (e.g. lowered mood and anxiety), stress associated with life circumstances (e.g. exams, meeting parental expectations, debt) and social isolation. This reportedly facilitated continued use:

*‘I know some of my friends [are] vaping or smoking because they are stressed and depressed … they heard nicotine or cigarettes can relieve some of [those symptoms].’* (20W, NS, NV)

*‘When I had my high school exams, it [vaping] was the main thing that I needed. The main reason why I’m not quitting now is because when I’m studying or work on something, it definitely calms me down.’* (19W, S, V)

*‘One of my friends told me, when he vapes, suddenly he said he’s happier…’* (20M, NS, NV)

### Addiction/dependence

Many participants described their friends’ and/or peers’ experiences of nicotine dependence. They spoke of frequent and increasing use, emotional and somatic symptoms associated with withdrawal, and relief from these symptoms after use:

*‘I have one somewhat good friend who tried to quit e-cigarettes, and he told me that he couldn’t sleep for weeks. He would wake up in sweats and shaking.’* (24M, S, V)

*‘I know like 50 percent of my friends … are addicted to it. But the [other] 50 they said they can control it.’* (20W, NS, NV)

No participants who used e-cigarettes reported being dependent or addicted to the devices themselves. Two participants believed that many young people lack an understanding of addiction and would not be able to identify if they were experiencing related symptoms:

*‘I think some people like to think that they’re in control. Like … I always say, “I’m not addicted”… I think it is something that they’re concerned about but just don’t necessarily understand as well.’* (19W, S, V)

## DISCUSSION

Results from this exploratory study investigating young adults’ experiences of psychological distress and e-cigarette use indicate a likely reciprocal relationship between mental health concerns and use whereby psychological distress in the form of low mood, anxiety, stress, and social isolation precipitates initiation, and use contributes to the maintenance of distress. Our results suggest that young people may lack an understanding of adaptive and maladaptive emotion regulation strategies and turn to e-cigarettes in a misguided attempt to manage distress. To address increases in e-cigarette initiation due to mental health concerns – and encourage cessation – public education on the potential for nicotine dependence to exacerbate stress and anxiety is warranted^16^. Given the use of e-cigarettes for stress management is a barrier to cessation^17^, young adults should be encouraged to use more adaptive coping strategies to support their mental health^18^. Attempts to encourage young adults to quit e-cigarettes should also be viewed in the context of mental health concerns to avoid negative outcomes.

The young adults in the present study discussed the presence of nicotine addiction and dependence among their peers but did not report experiencing this themselves. Interestingly, some participants believed that young people would not be able to identify symptoms of addiction, and this may have been occurring for participants in the present study who used e-cigarettes. Given young people’s apparent inability to self-identify nicotine addiction, those who use e-cigarettes should be provided with information on the early signs of dependence.

### Limitations

This work has several limitations. First, the sample size was small (although consistent with the qualitative nature of the study). Second, participants were students from one university, and findings may not be generalizable to all young adults. Finally, this study was conducted in Australia, and the results may not be generalized to other countries.

## CONCLUSIONS

To our knowledge, this study is the first in Australia to explore psychological distress and e-cigarette use among young adults and thus provides valuable insights into this relationship.

The findings should be treated as exploratory but do provide the impetus for a larger mixed-methods study to be conducted with a more diverse sample to understand the complexity of mental health and e-cigarette use. Research that investigates the role of adaptive coping strategies as potential protective factors against e-cigarette use is warranted.

## Supplementary Material



## Data Availability

The data supporting this research are available from the authors on reasonable request. Data will not be shared with those affiliated with the tobacco or e-cigarette industries.

## References

[1] Australian Institute of Health and Welfare. National Drug Strategy Household Survey 2022-2023: Electronic cigarettes and vapes. Accessed May 27, 2024. https://www.aihw.gov.au/reports/illicit-use-of-drugs/national-drug-strategy-household-survey/data

[2] Reifman A, Arnett JJ, Colwell MJ. Emerging adulthood: theory, assessment and application. Journal of Youth Development. 2007;2(1):37-48. doi:10.5195/jyd.2007.359

[3] Scales PC, Benson PL, Oesterle S, Hill KG, Hawkins JD, Pashak TJ. The dimensions of successful young adult development: a conceptual and measurement framework. Appl Dev Sci. 2016;20(3):150-174. doi:10.1080/10888691.2015.1082429 30344455 PMC6176765

[4] Jorm AF, Kitchener BA. Increases in youth mental health services in Australia: have they had an impact on youth population mental health? Aust N Z J Psychiatry. 2021;55(5):476-484. doi:10.1177/0004867420976861 33300364

[5] Gustavson K, Knudsen AK, Nesvåg R, Knudsen GP, Vollset SE, Reichborn-Kjennerud T. Prevalence and stability of mental disorders among young adults: findings from a longitudinal study. BMC Psychiatry. 2018;18(1):65. doi:10.1186/s12888-018-1647-5 29530018 PMC5848432

[6] Wingo AP, Baldessarini RJ, Windle M. Coping styles: longitudinal development from ages 17 to 33 and associations with psychiatric disorders. Psychiatry Res. 2015;225(3):299-304. doi:10.1016/j.psychres.2014.12.021 25582968 PMC4314401

[7] Bennett T, Holloway K. Motives for illicit prescription drug use among university students: a systematic review and meta-analysis. Int J Drug Policy. 2017;44:12-22. doi:10.1016/j.drugpo.2017.02.012 28343063

[8] Kenney SR, Anderson BJ, Stein MD. Drinking to cope mediates the relationship between depression and alcohol risk: different pathways for college and non-college young adults. Addict Behav. 2018;80:116-123. doi:10.1016/j.addbeh.2018.01.023 29407681 PMC5857241

[9] Jongenelis MI, Thoonen KAHJ. Factors associated with susceptibility to e-cigarette use among Australian adolescents. Int J Drug Policy. 2023;122:104249. doi:10.1016/j.drugpo.2023.104249 37918316

[10] Green VR, Conway KP, Silveira ML, et al. Mental health problems and onset of tobacco use among 12- to 24-year-olds in the PATH study. J Am Acad Child Adolesc Psychiatry. 2018;57(12):944-954.e4. doi:10.1016/j.jaac.2018.06.029 30522740 PMC7439766

[11] Wilson OWA, Bullen C, Duffey M, Bopp M. The association between vaping and health behaviors among undergraduate college students in the United States. J Am Coll Health. 2022:1-5. doi:10.1080/07448481.2022.2076097 35623030

[12] Becker TD, Arnold MK, Ro V, Martin L, Rice TR. Systematic review of electronic cigarette use (vaping) and mental health comorbidity among adolescents and young adults. Nicotine Tob Res. 2021;23(3):415-425. doi:10.1093/ntr/ntaa171 32905589

[13] Grant JE, Lust K, Fridberg DJ, King AC, Chamberlain SR. E-cigarette use (vaping) is associated with illicit drug use, mental health problems, and impulsivity in university students. Ann Clin Psychiatry. 2019;31(1):27-35. 30699215 PMC6420081

[14] Morgan DL, Nica A. Iterative thematic inquiry: a new method for analyzing qualitative data. International Journal of Qualitative Methods. 2020;19:1609406920955118. doi:10.1177/1609406920955118

[15] Braun V, Clarke V. Using thematic analysis in psychology. Qualitative Research in Psychology. 2006;3(2):77-101. doi:10.1191/1478088706qp063oa

[16] Moylan S, Jacka FN, Pasco JA, Berk M. Cigarette smoking, nicotine dependence and anxiety disorders: a systematic review of population-based, epidemiological studies. BMC Med. 2012;10:123. doi:10.1186/1741-7015-10-123 23083451 PMC3523047

[17] Dyson J, Bhatnagar M, Skinner J, Crooks M. Helping the quitters quit: a systematic review and narrative synthesis of the barriers and facilitators to e-cigarette cessation and the support that is needed. Patient Educ Couns. 2022;105(6):1402-1410. doi:10.1016/j.pec.2021.09.024 34579994

[18] Liu J, Gaiha SM, Halpern-Felsher B. A breath of knowledge: overview of current adolescent e-cigarette prevention and cessation programs. Curr Addict Rep. 2020;7(4):520-532. doi:10.1007/s40429-020-00345-5 33204602 PMC7661014

