# Supplementary material for: Psychological distress and e-cigarette use among young Australians: An exploratory, qualitative study
Source: Tob Induc Dis. 2024 Jun 19;22:10.18332/tid/189395. doi: 10.18332/tid/189395 (PMC11186307; doi:10.18332/tid/189395)
Supplement: Supplementary file 1 [file TID-22-114-s1.pdf]

References (image sources for Figure 1)

1. Sasek Z. Businessman Juggling, Time Management and Multitasking , Vector Cartoon Stick Figure Illustration. Canva, 2024.
2. Linear-design Images. Anxiety and Stress. Canva, 2024.
3. SAM Designs. Addiction Line Icon. Canva, 2024.
4. Circulon Tech. Coping Strategies Icon Style. Canva, 2024.

© 2024 Brierley M.E.E. et al.
